# Modular implementation of the linear- and cubic-scaling orbital minimization methods in electronic structure codes using atomic orbitals

**DOI:** 10.1098/rsos.230063

**Published:** 2023-04-26

**Authors:** Irina V. Lebedeva, Alberto García, Emilio Artacho, Pablo Ordejón

**Affiliations:** ^1^ CIC nanoGUNE BRTA, Donostia-San Sebastián 20018, Spain; ^2^ Catalan Institute of Nanoscience and Nanotechnology—ICN2 (CSIC and BIST), Campus UAB, Bellaterra 08193, Spain; ^3^ Simune Atomistics, Avenida de Tolosa 76, Donostia-San Sebastián 20018, Spain; ^4^ Institut de Ciència de Materials de Barcelona (ICMAB-CSIC), Bellaterra 08193, Spain; ^5^ Donostia International Physics Center DIPC, Donostia-San Sebastián 20018, Spain; ^6^ Theory of Condensed Matter, Cavendish Laboratory, University of Cambridge, Cambridge CB3 0HE, UK; ^7^ Ikerbasque, Basque Foundation for Science, Bilbao 48011, Spain

**Keywords:** density functional theory, linear-scaling methods, code modularization

## Abstract

We present a code modularization approach to design efficient and massively parallel cubic- and linear-scaling solvers for electronic structure calculations using atomic orbitals. The modular implementation of the orbital minimization method, in which linear algebra and parallelization issues are handled via external libraries, is demonstrated in the SIESTA code. The distributed block compressed sparse row (DBCSR) and scalable linear algebra package (ScaLAPACK) libraries are used for algebraic operations with sparse and dense matrices, respectively. The MatrixSwitch and libOMM libraries, recently developed within the Electronic Structure Library, facilitate switching between different matrix formats and implement the energy minimization. We show results comparing the performance of several cubic-scaling algorithms, and also demonstrate the parallel performance of the linear-scaling solvers, and their supremacy over the cubic-scaling solvers for insulating systems with sizes of several hundreds of atoms.

## 1. Introduction

The success of electronic structure theory [1] in modelling new materials and devices [2,3] has stimulated the development of hundreds of electronic structure codes [4]. Historically almost all of these individual software packages are written in distinct ways, although many tasks performed are similar. Except for numerical and performance-related libraries such as basic linear algebra subroutines (BLAS) [5], higher-level linear algebra utilities (serial linear algebra package—LAPACK [6] and its parallel counterpart, scalable LAPACK—ScaLAPACK [7]), message passing interface (MPI) level [8] etc., significant parts of the codes are replicated with some variations. Electronic structure packages are growing rapidly, incorporating more and more new features. Also, the codes have to adapt to the constant hardware evolution, which in the case of monolithic code architecture implies significant efforts on re-engineering. In this situation, it seems more efficient to change the traditional monolithic paradigm of software development to the modular one in which common tasks arise [9].

In addition, such an approach allows to separate the tasks related to high-level routines focused on the calculation of physical properties from the implementation of the underlying routines for parallelization and algebra. On one hand, this means that the implementation of new models and algorithms becomes much simpler and does not require the knowledge of technical details related to parallelization. On the other hand, much better performance is achieved using specialized external libraries, and thus much larger systems can be modelled. Significant efforts (e.g. the European project MAX [10]) are underway to stimulate the paradigm change in software design and to facilitate exascale computing. Here, we show the benefits of modularization by the example of SIESTA [11–15].

SIESTA [11–15] was specifically designed for linear-scaling calculations [16,17] in which the computational time grows linearly with the number of atoms [16–21]. Such methods make possible calculations of large systems at a considerably less computational cost compared with common cubic-scaling approaches. SIESTA uses strictly localized atomic-like functions for basis sets in which the Hamiltonian and overlap matrices, **H** and **S**, are sparse. If, additionally, the confinement of the wave functions is imposed, the coefficient matrix **C** expanding wave functions in the basis is also sparse. Reducing the problem of solving the Kohn–Sham equations to the minimization of a properly constructed energy functional within the Ordejón–Mauri [16–18,22] and Kim [23] approaches, the inversion of the overlap matrix is avoided and only expressions involving products and sums of sparse **H**, **S** and **C** matrices need to be computed, all in linear-scaling effort.

**Figure 1.**
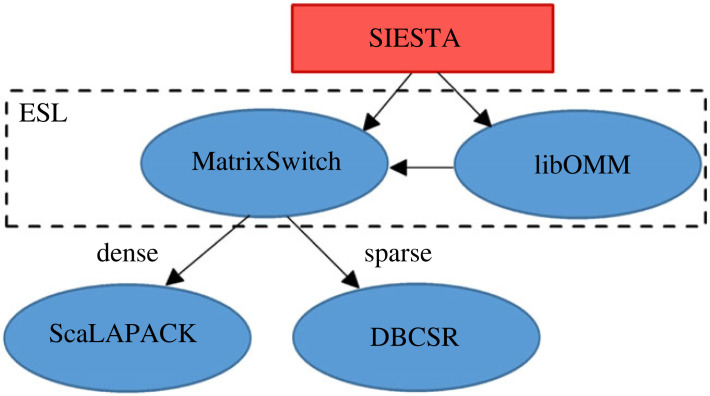
The use of libraries within the revised orbital minimization method (OMM) solver in the electronic structure code SIESTA [11–15]. The red rectangular box corresponds to SIESTA. Blue ellipses indicate the libraries used [7,9,24,25,27–30]. The libraries in the dashed frame belong to the Electronic Structure Library (ESL) [9,31]. The arrows demonstrate calls to the libraries.

The linear-scaling solvers in SIESTA, although available from the start [12], are not widely used in practice. One of the reasons is that the implementation of these physical methods involved also coding of the algebra and parallelization of sparse matrices, which inevitably increased the code complexity and hindered progress. Recent efforts on linear-scaling methods have produced the distributed block compressed sparse row (DBCSR) library [24] that efficiently handles algebraic operations for sparse matrices and is massively parallelized [25,26]. Using this library, we have implemented an improved and more reliable version of linear-scaling solvers in SIESTA (figure 1).

Another recent initiative that has helped re-designing SIESTA is the Electronic Structure Library (ESL) [9,31], a collaboration platform for shared software development. We use ESL’s libOMM library [9,27] distributed within the omm-bundle [32]. It encodes the Ordejón–Mauri [16–18,22] and Kim [23] functionals, originally without the additional approximation of wave function confinement, rendering dense **C** matrices and cubic scaling. Such an approach provides an alternative to conventional cubic-scaling methods, which can be faster in long simulations by avoiding computationally expensive orthonormalization and using history on previous steps [28]. We refer to unconstrained minimization methods of suitable energy functionals, with either linear or cubic scaling, as the orbital minimization method (OMM) [21,28,33,34]. In libOMM [9,27,28], the minimization is customarily performed via conjugate gradients (CG). The parameters of the quartic function describing the energy dependence along the search direction are computed analytically [17,28].

Although the original libOMM library provides cubic scaling [9,27,28], it has been straightforward to extend it to linear scaling, the equations being almost the same, the key difference being the use of sparse matrices instead of dense. Normally two separate pieces of the code dealing with sparse and dense matrices would be used for the same equations. This code duplication can be avoided in libOMM thanks to the MatrixSwitch (MS) library [29], an interface between high-level physical routines and low-level routines for matrix algebra. MS, which is also distributed within the omm-bundle [32] of ESL [9,31], simplifies the coding of matrix operations and allows a single code independent of matrix format, by means of format-independent high-level commands. Depending on matrix format, MS calls the appropriate linear algebra library. An example of calculations using the MS library is shown in listing 1 (see electronic supplementary material for MS overview).

Recently, the MS library was extended to support sparse matrices [9,30] via the DBCSR [24–26] library. Here, we consider dense and sparse matrices in the parallel-distributed dense block cyclic (*pddbc*) and parallel-distributed compressed sparse row (*pdcsr*) MS formats for which algebraic operations are handled with the help of the ScaLAPACK [7] and DBCSR [24] libraries, respectively. Although basic functionality for sparse matrices was already provided in this recent MS version [9,30], a revision of the library was needed towards treating sparse and dense matrices on the same footing, and to enable linear-scaling calculations. The incorporation of the solver library into an electronic structure code also implies additional matrix manipulations such as conversions between the matrix formats supported by the code and the solver library as well as reading and writing of restart files. The corresponding subroutines have been here implemented in MS and are discussed below.

After a brief overview of the OMM approaches, the new implementation of linear- and cubic-scaling OMM in SIESTA is presented, including the necessary changes in the MS and libOMM libraries forming part of ESL. The results of the first tests are discussed, and recommendations on the efficient use of OMM are given.

**Listing 1.**
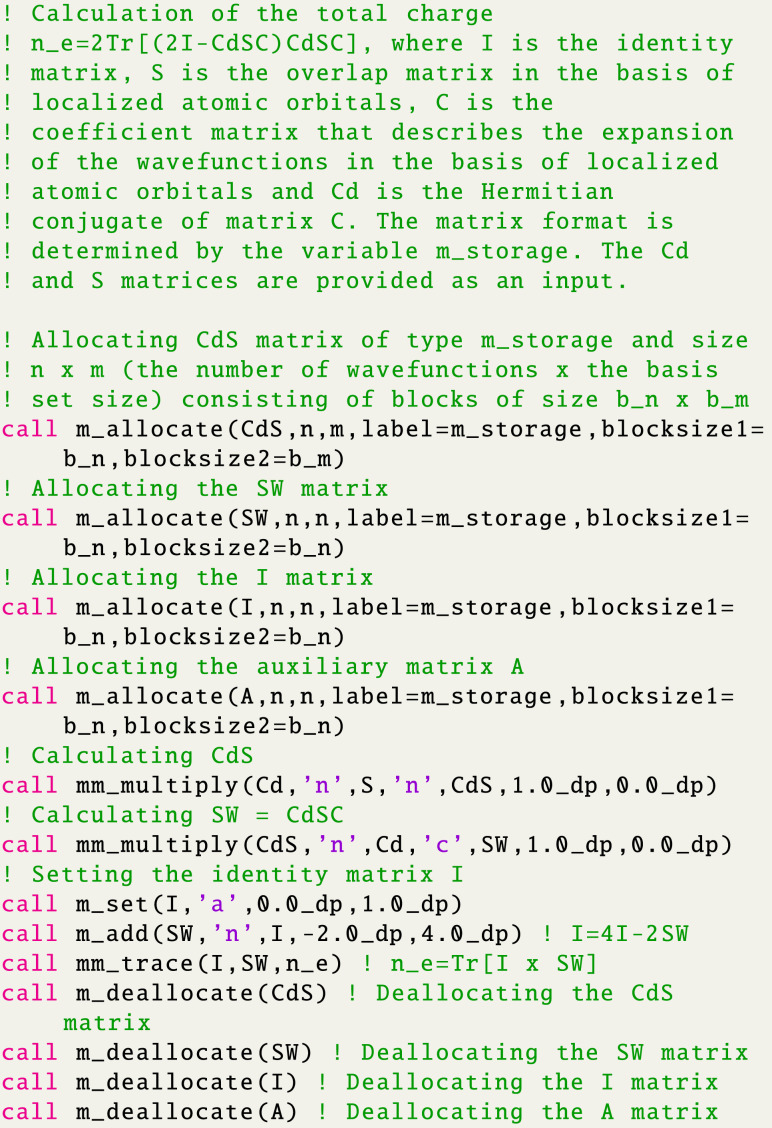
An example of the calculation of the total charge in the OMM approach using the MatrixSwitch library.

## 2. Overview of orbital minimization method approaches

In density functional theory (DFT) [35,36], the problem of finding the ground state of a many-electron system is reduced to an energy minimization for the system of 2*n* non-interacting electrons moving in an effective potential and described by one-particle states {|*ψ*_*i*_〉} (*i* = 1, …, *n*) each of which is occupied by two electrons of opposite spin (assuming no spin polarization, for simplicity). The set of states {|*ψ*_*i*_〉} is one of the many possible bases in the occupied subspace of the Hilbert space of the system and can be chosen orthonormal or not. In the latter case [37], the overlap matrix **S_W_** with the elements (*S*_*W*_)_*ij*_ = 〈*ψ*_*i*_|*ψ*_*j*_〉 is not the identity matrix (**S_W_** ≠ **I**, (*I*_*W*_)_*ij*_ = *δ*_*ij*_) and the density matrix operator that determines the projection onto the occupied subspace is then given by

2.1 $$\hat{\rho }=2\sum_{i,j=1}^{n}|\psi_{i}\rangle {\left({S_{W}}^{-1}\right)}_{ij}\langle \psi_{j}|,$$

involving the inverse of **S_W_** [17,18,22]. Here and below, we limit our consideration to insulating systems. The linear-scaling methods applicable to metals are discussed e.g. in [38–41].

The corresponding band structure energy becomes [16–18,22]

2.2 $$E=\mathrm{Tr}[\hat{H}\hat{\rho }]=2\mathrm{Tr}[{S_{W}}^{-1}H_{W}],$$

where $\hat{H}$ is the Hamiltonian operator, and **H_W_** is the matrix with the elements ${\left(H_{W}\right)}_{ij}=\langle \psi_{i}|\hat{H}|\psi_{j}\rangle$. Note that the traces in equation (2.2) are taken on spaces of different dimensions: the size of the basis set for the first, and of the occupied states in the second. Also, the second equality holds for zero temperature.

In the basis of *m* functions {|*ϕ*_*i*_〉} (strictly localized atomic orbitals in SIESTA)

2.3 $$|\psi_{i}\rangle =\sum_{\mu =1}^{m}C_{i}^{\mu }|\phi_{\mu }\rangle ,$$

where we refer to **C** as the coefficient matrix. Then $H_{W}=C^{†}HC$ and $S_{W}=C^{†}SC$, where $H_{ij}=\langle \phi_{i}|\hat{H}|\phi_{j}\rangle$, *S*_*ij*_ = 〈*ϕ*_*i*_|*ϕ*_*j*_〉 and $C^{†}$ is the Hermitian conjugate of **C**. The energy functional in equation (2.2) is minimized to find the ground state energy. The most common approach is direct diagonalization of the Hamiltonian matrix **H** (an *m* × *m* matrix for the basis set of size *m*). Energy and charge density are then obtained using the wave functions and energies of the *n* lowest eigenstates. By contrast, in the iterative approaches [42], the energy is minimized with respect to variations in the states {|*ψ*_*i*_〉}. Here one needs to calculate the inverse of the overlap matrix **S_W_**^−1^ or impose the orthonormality condition (*S*_*W*_)_*ij*_ = *δ*_*ij*_. In any case, the computational time increases as *O*(*n*^3^) with the system size, while the memory required to store the wave functions grows as *O*(*n*^2^).

In OMM approaches [16–18,22], the expensive orthonormalization step is avoided via the modification of the energy functional in such a way that it automatically induces the orthonormalization of the wave functions during minimization

2.4 $$\begin{array}{rl} \tilde{E} & =2\mathrm{Tr}[(I_{W}+(I_{W}-S_{W}))H_{W}]\\ & =2\mathrm{Tr}[(2I_{W}-C^{†}SC)C^{†}HC]. \end{array}$$

This expression can be derived from consideration of Lagrange multipliers [16,17] or expansion of the inverse overlap matrix to first order in the deviation from the identity [18,22]: **S_W_**^−1^ ≈ **I_W_** + (**I_W_** − **S_W_**). The solution obtained from equation (2.4) is the same as from equation (2.2).

Within the same approximation, the density matrix of equation (2.1) is computed as [17]

2.5 $$\rho =C(I_{W}+(I_{W}-S_{W}))C^{†}=2C(2I_{W}-C^{†}SC)C^{†}$$

and the forces on atom *I* as [17]

2.6 $$F_{I}=-\mathrm{Tr}\left[\rho \frac{\partial H}{\partial R_{I}}\right]+\mathrm{Tr}\left[\rho_{E}\frac{\partial S}{\partial R_{I}}\right],$$

where we refer to $\rho_{E}=2CH_{W}C^{†}$ as the ‘energy density’.

If the basis functions and wave functions are chosen to be strictly localized, the Hamiltonian, overlap and coefficient matrices, **H**, **S** and **C**, are sparse and *O*(*n*) scaling with system size is achieved [16–18,22]. Note that this is not the case for equations (2.1) and (2.2) as the inverse of **S** is not sparse (although sub-cubic scaling can be achieved using selected inversion to compute just the needed elements of the inverse [43]). In the case of periodic systems, localized wave functions are close to the Wannier functions that decay exponentially with the distance from the centre of localization in insulators and in metals at a finite temperature. Imposing localization constraints on the wave functions, however, leads to a deviation from the exact solution of equations (2.2) and (2.4). Also the localized wave functions obtained are not strictly orthonormal and do not comply with the system symmetries [23]. However, the degree of approximation can be controlled with the cut-off radius *R*_*C*_ for the wave functions. Here, we limit our consideration to insulators with a substantial band gap, where *R*_*C*_ of several Å is normally enough [17,18].

In the Ordejón–Mauri functional [16–18,22], the localization of the wave functions gives rise to many shallow local minima and flat regions in which the algorithm can be trapped for a long time during the energy minimization. This problem is solved in the Kim functional [23] by including unoccupied states and introducing a chemical potential *η*, i.e. the energy separating occupied and unoccupied states. The corresponding functional is obtained by (i) an eigenspectrum shift **H** → **H** − *η***S**, (ii) changing dimensions of **C** from *m* × *n* to *m* × *n*^′^, where *n*^′^ > *n*, and (iii) changing the energy functional in equation (2.4) as $\tilde{E}\to \tilde{E}+\eta n$, and energy density ***ρ***_*E*_ in equation (2.6) as ***ρ***_*E*_ → ***ρ***_*E*_ + *η****ρ***. It should be noted, however, that although the multiple-minima problem is solved in the Kim functional, it is sometimes hard to choose a proper value for *η*. It should always lie within the band gap, but the bands can move up and down during self-consistency or molecular dynamics (MD), *η* possibly getting into the valence or conduction bands and, as a result, converging to an erroneous solution. Care should be taken to ensure that the solution reproduces the correct number 2*n* of electrons.

If the localization constraints on the wave functions are removed, the exact solution of equations (2.2) and (2.4) is obtained [28]. Even in this case, however, one energy minimization can demand many CG iterations. This relates to the problem of length-scale or kinetic energy ill-conditioning [42,44]. The efficiency of the CG algorithm depends on the ratio of the maximal and minimal extremal curvatures of the function minimized, which in OMM are determined by the maximal and minimal eigenvalues of the Hamiltonian. The eigenspectrum of the Hamiltonian is broad, given the large kinetic energy of high-energy eigenstates. Although such states contribute negligibly to the ground state solution, the problem becomes ill-conditioned and the convergence is slow. It is, however, possible effectively to reduce the width of the eigenspectrum by suppressing the kinetic energy contribution of high-energy states through preconditioning [45,46], by which the CG gradient matrix is multiplied by the preconditioning matrix [28]

2.7 $$P={\left(S+\frac{T}{\tau_{T}}\right)}^{-1},$$

where *τ*_*T*_ is the scale for kinetic energy preconditioning and **T** is the kinetic energy matrix. Another approach for improving the efficiency of CG minimizations is reducing the generalized eigenvalue problem to the standard form via the Cholesky factorization [28]. Both of these approaches involve matrices that are not sparse (the preconditioning matrix or the reduced Hamiltonian) and are considered here only for cubic-scaling OMM.

## 3. Modular solver architecture

### 3.1. Solver input and output

**Figure 2.**
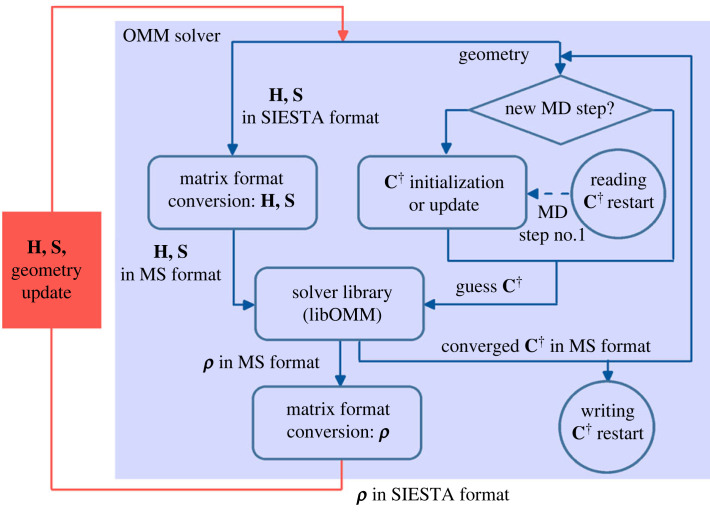
Scheme of the revised OMM solver. Blocks within the solver are shown in blue. The rest of the SIESTA code is shown as a red block. Arrows indicate data flow. Hamiltonian, overlap and density matrices are denoted as **H**, **S** and ***ρ***. The Hermitian conjugate of the coefficient matrix of expansion of the wave functions in the basis of localized atomic orbitals is denoted as $C^{†}$. The Hamiltonian and overlap matrices are converted from internal SIESTA to MatrixSwitch (MS) format for further calculation of ***ρ*** and $C^{†}$ with the help of the libOMM library. The restart file for $C^{†}$ can be read once at the first molecular dynamics (MD) step.

A scheme of the implemented OMM solver is shown in figure 2. At each self-consistent-field (SCF) step, the solver receives as an input the Hamiltonian and overlap matrices in the basis of strictly localized atomic orbitals, **H** and **S**, and the information on the system geometry. SIESTA uses for matrices the standard compressed sparse row format, that is the matrix information is stored in local one-dimensional arrays containing data values and column indices for individual non-zero elements of local rows as well as indices of the first non-zero elements and numbers of non-zero elements for each local row. The blocks of rows are distributed on a one-dimensional process grid (figure 4*a*). Here and in MS we refer to this format as *pdrow* to distinguish from the *pdcsr* format supported by DBCSR. **H** and **S** are received by the solver in the *pdrow* format. The density matrix ***ρ*** is the output, also in *pdrow* (see equation (2.5)). This matrix is used to update **H** for the next SCF step outside the solver. At the end of each MD step, the solver is called again to compute the energy density matrix ***ρ***_*E*_, which, along with ***ρ***, is later used to calculate forces (see equation (2.6)) and stresses. The scheme of the ***ρ***_*E*_ calculation is analogous to that of ***ρ*** shown in figure 2.

### 3.2. Solver library

The solver uses the libOMM library [9,27,28,32] to perform the CG minimization of the energy functional given by equation (2.4). As an input, the libOMM library requires **H** and **S**, as well as the initial guess for $C^{†}$, in one of the MS formats [9,29,30,32]. As an output, it provides the converged $C^{†}$, and ***ρ*** or ***ρ***_*E*_ in the same format. The *pddbc* format is used for parallel calculations with dense matrices. In this case, all matrix elements are stored and algebraic operations are performed using the ScaLAPACK library [7]. The matrix is divided into two-dimensional blocks distributed on a two-dimensional or one-dimensional process grid. For parallel calculations with sparse matrices, the *pdcsr* format is used. The matrix is also divided into two-dimensional blocks distributed on a one-dimensional or two-dimensional process grid (figures 4*b*,*c*, respectively). However, in this case, zero blocks are not stored. The algebraic operations are performed by the DBCSR library [24–26]. At the moment, libOMM supports only equal rectangular blocks.

The equations implemented in the libOMM library are compatible with all OMM flavours discussed in the previous section, including the Ordejón–Mauri and Kim functionals, with and without localization constraints. However, to make the libOMM library functional for sparse matrices, some parts to the code have been reformulated. Now block-size information is passed to the MS library during the allocation of intermediate matrices required for the CG minimization using m_allocate() (see electronic supplementary material). Also, sparsity is imposed on the gradient matrix **G** (with the elements $G_{i}^{\mu }=\partial \tilde{E}/\partial {\left(C_{i}^{\mu }\right)}^{*}$) following the sparsity pattern of the initial guess for **C**. Already during **G** calculation [28], only matrix elements that fit into the sparsity pattern are computed in the contributions to **G** that are given by products of matrices (using keep_sparsity = true option of mm_multiply()). In the rest of the contributions, non-zero elements that do not fit into the sparsity pattern are omitted and no longer stored, while zero elements within the sparsity pattern are stored as zeros. The sparsity of the density (***ρ***) and energy density (***ρ***_*E*_) matrices is assumed to be the same as of the overlap matrix **S** and only elements of these matrices that fit into the sparsity pattern are computed. Additionally, the expression for the calculation of ***ρ***_*E*_ has been corrected as compared with the previous libOMM version [28] in accordance with equations (2.4)–(2.6) and [17]. The Cholesky factorization and kinetic energy preconditioning are available only for dense matrices.

### 3.3. $C^{†}$ matrix format conversion

**Figure 3.**
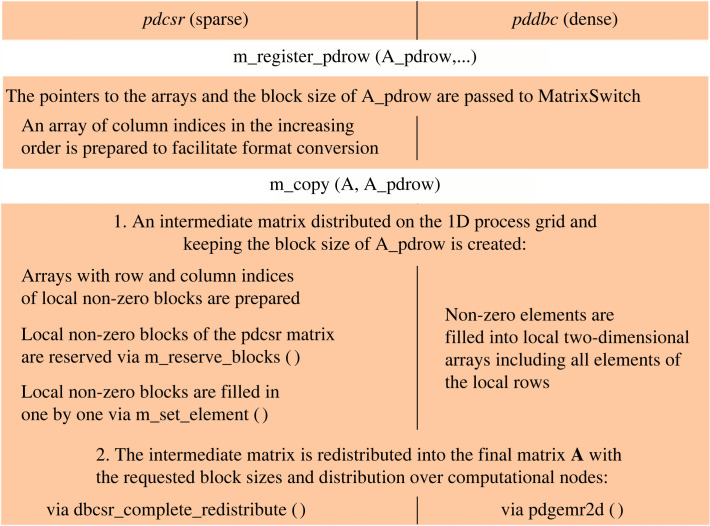
A series of calls to the MatrixSwitch library required for the format conversion of matrix **A** from the *pdrow* format used in SIESTA (A_pdrow) to the *pdcsr* and *pddbc* MatrixSwitch formats (**A**) handled with the DBCSR and ScaLAPACK libraries, respectively.

**Figure 4.**
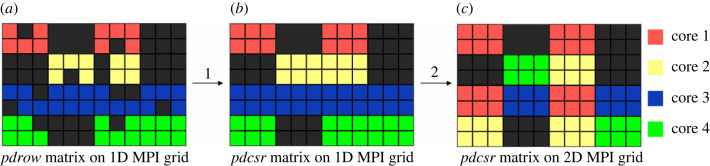
Example of matrix format conversion from the *pdrow* format used in SIESTA to *pdcsr* MatrixSwitch format handled with DBCSR: (*a*) *pdrow* matrix distributed on the one-dimensional process grid with four CPU cores, (*b*) *pdcsr* matrix with 2 × 3 blocks distributed on the same one-dimensional process grid and (*c*) *pdcsr* matrix with 2 × 3 blocks distributed on the 2 × 2 two-dimensional process grid. Arrows indicate steps 1 and 2 of subroutine m_copy() as explained in figure 3. Small squares represent elements of the 8 × 12 matrix. Black squares are zero elements that are not stored. Red, yellow, blue and green squares correspond to elements stored on cores 1, 2, 3 and 4, respectively.

In order to incorporate the libOMM library into SIESTA within the OMM solver, the following steps are required (figure 2): (1) matrix format conversion from/to the SIESTA format to/from the MS formats and (2) initialization and update of $C^{†}$, according to the current geometry of the system. The matrix format conversion is realized using calls to MS subroutines m_register_pdrow() and m_copy() (see electronic supplementary material). The first of this subroutines has been added to the MS library and the second one has been extended to allow the conversion from/to the *pdrow* format to/from the *pdcsr* and *pddbc* formats. The conversion is performed as follows (figure 3). First the pointers to arrays of the *pdrow* matrix and its block size are passed to MS. Then a *pdcsr*/*pddbc* matrix distributed on the one-dimensional process grid with the same block size for rows as the initial *pdrow* matrix is filled in element by element (figure 4). The missing elements of the *pddbc* matrix or within non-zero blocks of the *pdcsr* matrix are filled with zeros. Note that to speed up the conversion and guarantee linear scaling, column and row indices of non-zero blocks of the *pdcsr* matrix should be passed to the DBCSR library before filling the values via the call to m_reserve_blocks() (see electronic supplementary material). Once the one-dimensional-distributed *pdcsr*/*pddbc* matrix is ready, it can be redistributed on a two-dimensional process grid. In the case when the final matrix is distributed on the one-dimensional process grid and has the same block size for rows at the initial *pdrow* matrix, the last step is omitted.

The conversion from *pdcsr* and *pddbc* to *pdrow* is implemented in a similar way. It is assumed that the row and column indices of non-zero elements of the *pdrow* matrix are already known. Only the values of the matrix elements are restored.

### 3.4. $C^{†}$ matrix initialization and update

The initialization of the $C^{†}$ matrix in the sparse form is performed in SIESTA in the following way. It is supposed that each atom carries the number of localized wave functions equal to the atomic charge (in units of elementary charge) divided by two, *Q*_at_/2. If *Q*_at_ is odd, (*Q*_at_ + 1)/2 localized wave functions are assigned to one atom and (*Q*_at_ − 1)/2 to the next one. This procedure is repeated for all the atoms in the system. Then the $C^{†}$ matrix in the *pdrow* format with the total number of rows that corresponds to the total number of localized wave functions, *N*_WF_ = *Q*/2, where *Q* is the sum of atomic charges in the system, is prepared. The local rows are assigned according to the block size *b*_WF_. By default, it equals the block size for the basis functions, *b*_BF_, multiplied by the ratio of the total number *N*_WF_ of localized wave functions to the basis set size *N*_BF_: *b*_WF_ = *b*_BF_*N*_WF_/*N*_BF_. For each local row, the local environment of the atom hosting the corresponding localized wave function is analysed. The row elements that correspond to atoms beyond some cut-off radius *R*_*C*_ from the atom considered are supposed to be zero. The row elements that correspond to atoms within the cut-off radius *R*_*C*_ are initialized by random values. This sparsity pattern is maintained during the energy functional minimization. The $C^{†}$ matrix in the *pdrow* format is converted to the *pdcsr* or *pddbc* formats in the same manner as the Hamiltonian and overlap matrices, **H** and **S**.

It should be mentioned also that the initial cut-off radius *R*_*C*,ini_ for initialization of the $C^{†}$ matrix can be set different from *R*_*C*_ used for the energy minimization. Choosing a small initial radius *R*_*C*,ini_ (several Å) helps to avoid convergence problems and is useful not only in calculations with sparse matrices but also with dense ones.

At each new MD step, the sparsity pattern of the $C^{†}$ matrix is checked again. The elements that now should be zero because the corresponding atoms got away by more than *R*_*C*_ are set to zero and no longer stored. The elements corresponding to the atoms that got closer than *R*_*C*_ are now stored and treated as non-zero but are assigned to zero as the initial guess. Linear extrapolation of the $C^{†}$ matrix based on the information from the two previous MD steps is also possible.

### 3.5. $C^{†}$ matrix input and output

The restart file for the $C^{†}$ matrix can be written at each SCF step and read at the beginning of the run. These operations are performed by calling new MS subroutines m_read() and m_write(), respectively (see electronic supplementary material). If the $C^{†}$ matrix in the *pdcsr* or *pddbc* format is distributed on a two-dimensional process grid, it is first converted into a one-dimensional-distributed matrix (by analogy with the format conversion routines). Then the blocks of rows are consecutively passed to the head core and written to the file. To read the file, the reverse operations are performed. The block sizes and process grid for the $C^{†}$ matrix do not need to be the same as used when writing the restart information. Upon reading, the sparsity pattern of the $C^{†}$ matrix is corrected according to the current system geometry.

### 3.6. SIESTA input parameters

The input parameters for SIESTA corresponding to the revised OMM solver are described in table 1. To use the OMM solver, SolutionMethod should be set to BLOMM (OMM with block matrices).

**Table 1.** Principal input parameters for the revised OMM solver in SIESTA (SolutionMethod BLOMM) and their default values.

input parameter	default value	description
OMM.UseSparse	true	whether to use sparse matrices
OMM.UseKimFunctional	true	whether to use the Kim [23] (or Ordejón–Mauri [16–18,22]) functional
OMM.Use2D	true	whether to distribute matrices on a two-dimensional process grid
OMM.ReadCoeffs	false	whether to read the initial localized wave functions (LWFs), i.e. the $C^{†}$ matrix, from the restart file (*.WF_COEFFS_BLOMM)
OMM.WriteCoeffs	false	whether to write the LWFs ($C^{†}$ matrix) to the restart file
OMM.RelTol	10^−9^	the tolerance for the energy convergence in conjugate-gradient (CG) iterations. When 2(*E*_*n*_ − *E*_*n*−1_)/(*E*_*n*_ + *E*_*n*−1_), where *E*_*n*_ is the energy at CG iteration *n*, becomes smaller than this tolerance, CG iterations are stopped
OMM.BlockSizeC	*b*_WF_ = *b*_BF_*N*_WF_/*N*_BF_	the block size for LWFs (rows of the $C^{†}$ matrix). By default, equals the block size for the basis functions *b*_BF_ (input parameter BlockSize) multiplied by the ratio of the total number *N*_WF_ of LWFs to the basis set size *N*_BF_
OMM.Eta	0 eV	the chemical potential for the Kim functional
OMM.RcLWF	9.5 Bohr	the cut-off radius *R*_*C*_ for LWFs determining the sparsity pattern of the $C^{†}$ matrix
OMM.RcLWFInit	0 Bohr	the initial cut-off radius *R*_C__,ini_ for LWFs. It is the same as OMM.RcLWF if set to 0
OMM.Extrapolate	false	whether to estimate LWFs at the next molecular dynamics (MD) step by the linear extrapolation of the results of two last MD steps
only for the cubic-scaling OMM		
OMM.Precon	-1	the number of self-consistent-field (SCF) steps for which to apply the preconditioning [28]. If negative, the preconditioning is applied at all SCF steps
OMM.PreconFirstStep	OMM.Precon	OMM.Precon for the first MD step
OMM.TPreconScale	10 Ry	the scale *τ*_*T*_ for the kinetic energy preconditioning (see equation (2.7))
OMM.Cholesky	false	whether to apply the Cholesky factorization [28]

## 4. Tests

### 4.1. Computational details

The test calculations have been carried out for single-layer boron nitride (BN) under periodic boundary conditions. Supercells of BN from 12 × 12 to 96 × 96 with up to 18 400 atoms are considered. The lattice constant is set at 2.48 Å. The height of the simulation cell is 20 Å. The calculations have been performed at the single Γ point. The local density approximation [47], norm-conserving Troullier–Martins [48] pseudopotentials and standard built-in double-zeta polarized (DZP) basis set [49] are used. The atomic orbitals are set to zero beyond the cut-off determined by the energy shift of 10 meV (cut-off radii 2.5–4.5 Å). The real-space grid is equivalent to the plane-wave cut-off energy of 100 Ry. The linear mixing scheme with a mixing parameter of 0.1 is applied to converge the ground state. The tolerance is 10^−4^ for the density matrix and 10^−3^ eV for the matrix elements of the Hamiltonian.

To test performance of different approaches in MD simulations, several MD steps starting from the converged ground state have been computed (the ground state is converged previously with the same method as used for MD). The microcanonical ensemble with an initial temperature of 300 K is considered. The Verlet algorithm [50] with a time step of 1 fs is used. The Pulay mixing scheme [51] with a mixing parameter of 0.2 is applied during the MD simulations.

The matrices involved in the calculations consist of equal blocks. For the DZP basis set, each boron and nitrogen atom has 13 basis functions, and hosts three or two wave functions depending on whether the unoccupied states are included into consideration or not, respectively. Therefore, the block size for the wave functions is usually chosen to be *b*_WF_ = 6 and for the basis functions *b*_BF_ = 13. The matrices are distributed on a two-dimensional process grid. The cut-off radius for localized wave functions in typical calculations with sparse matrices is *R*_*C*_ = 4 Å. The chemical potential for the Kim functional is *η* = −5.5 eV. CG iterations are performed until the difference of energies at consecutive CG iterations divided by the average energy at these iterations reaches 10^−9^. The tests with the preconditioning for dense matrices have been carried out using the scale for the kinetic energy of *τ*_*T*_ = 10 Ry [28].

### 4.2. Results

**Figure 5.**
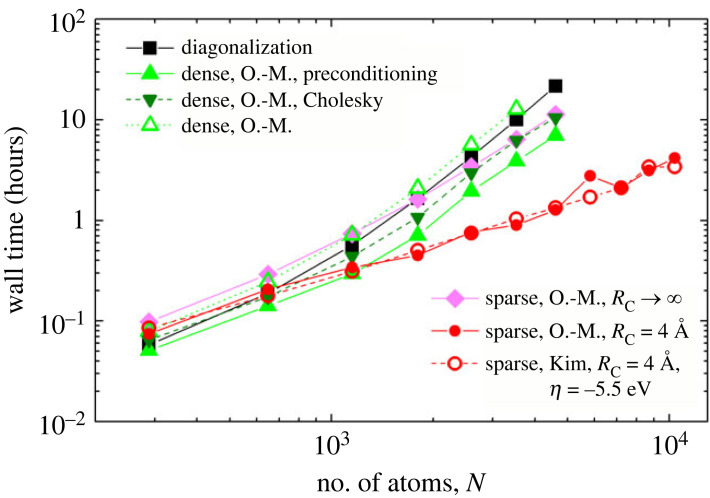
Wall time (in hours) for four MD steps for a single boron nitride (BN) layer computed using different approaches versus number *N* of atoms in the system: (black squares) diagonalization, (green triangles up) OMM with dense matrices (using ScaLAPACK) and preconditioning using a kinetic-energy scale *τ*_*T*_ = 10 Ry, (dark green triangles down) OMM with dense matrices with Cholesky factorization (open green triangles up) plain OMM with dense matrices, (magenta diamonds) OMM with sparse matrices (using DBCSR) without wave function localization (wave function cut-off radius *R*_*C*_ → ∞), (red circles) Ordejón–Mauri functional with *R*_*C*_ = 4 Å and (open red circles) Kim functional with *R*_*C*_ = 4 Å and chemical potential *η* = −5.5 eV. In all the cases without wave function localization, the Ordejón–Mauri functional is considered. The calculations are performed on 96 CPU cores. A double-zeta polarized (DZP) basis set is used. The block size is *b*_WF_ = 6 for the wave functions and *b*_BF_ = 13 for the basis functions.

To compare the performance of diagonalization and OMM with dense and sparse matrices, we have performed test MD simulations for single-layer BN in different sizes. Figure 5 demonstrates that the approaches in which the wave functions are not confined in space have much worse scaling with system size than the methods with localized wave functions within a cut-off radius *R*_*C*_. The scaling of the former approaches is close to cubic for large systems (exceeding 1000 atoms in our calculations). It should be noted, however, that for small systems (within 1000 atoms) the scaling is sub-cubic. The reason is that for such systems the solver contribution to the total time plotted in figure 5 is comparable to the contributions of other parts of the code that have linear scaling with system size. Among the methods using dense matrices, OMM with applied preconditioning or Cholesky factorization, which improve convergence, shows a slightly better scaling compared with diagonalization or plain OMM. Also OMM using the DBCSR library with no localization of wave functions (*R*_*C*_ → ∞) clearly has a better scaling than OMM using ScaLAPACK. This is explained by the fact that the former, although having a dense coefficient matrix, still exploits the sparsity of the Hamiltonian and overlap.

In the range of system sizes considered, OMM with kinetic energy preconditioning is the fastest among the approaches without wave function localization, followed by OMM with the Cholesky factorization, diagonalization and plain OMM (figure 5). The crossover between preconditioned dense OMM and the linear-scaling methods takes place for the system with about 1200 atoms. For the plain dense OMM and for diagonalization, the crossovers with linear-scaling methods occur earlier, at about 300 and 700 atoms, respectively.

**Figure 6.**
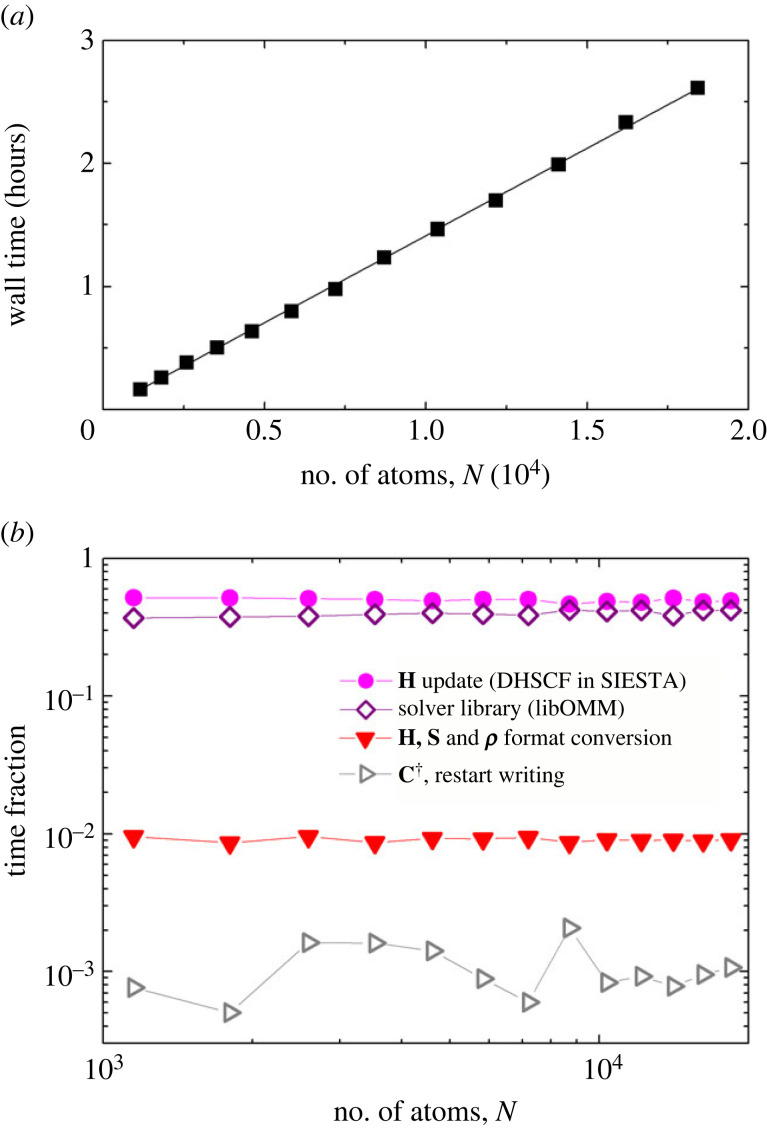
(*a*) Wall time (in hours) for three MD steps with 12 SCF iterations each for a single BN layer described using the Kim functional versus number *N* of atoms (in 10^4^ atoms). A linear fit is shown by the solid line. (*b*) Relative contributions of the OMM solver subroutines to the total time versus *N*: (magenta circles) Hamiltonian update on a three-dimensional grid (DHSCF subroutine in SIESTA [13]), (open purple diamonds) solver library libOMM, (red triangles down) format conversion of **H**, **S** and ***ρ*** and (open grey triangles right) writing restart for localized wave functions ($C^{†}$). The calculations are performed on 192 CPU cores for a DZP basis, *R*_*C*_ = 4 Å, *η* = −5.5 eV, *b*_WF_ = 6 and *b*_BF_ = 13.

Our timings for single-layer BN have confirmed that the Ordejón–Mauri and Kim approaches in which the wave functions are localized within a cut-off radius *R*_*C*_ show linear scaling with system size (figure 6*a*). The computational times corresponding to different parts of the solver (matrix conversion, libOMM library, initialization and update of the coefficient matrix, reading and writing of restart for localized wave functions) and other parts of the SIESTA code such as the subroutine for the Hamiltonian update called after the density matrix change at each SCF step (DHSCF), all do change linearly upon increasing the system size. As a result, relative contributions of different parts of the code do not depend on the system size (figure 6*b*). This is different from the cubic-scaling methods, in which the solver very early takes most of the computing time upon increasing the system size, since the rest of the code has linear scaling. It should also be noted that, for the system considered, the solver takes only 40–50% of the computational time, comparable, for example, to the subroutine for the Hamiltonian update (DHSCF in SIESTA). Most of this time corresponds to the minimization of the energy functional given by equation (2.4) performed by the solver library libOMM. The matrix format conversion takes only 0.5–1.0% of the total time. Writing of the restart files for localized wave functions takes up to 0.3% of the time, and initialization and update of the coefficient matrix take a negligible time within 0.01%.

**Figure 7.**
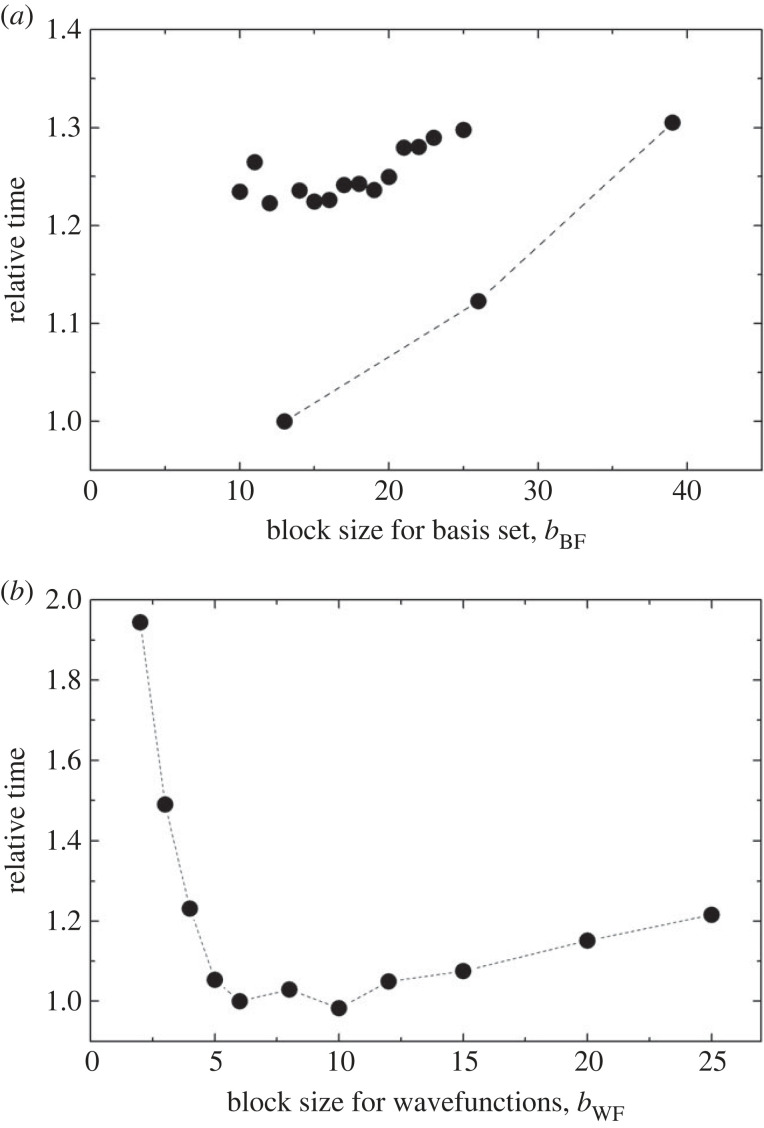
Relative time for the solver library libOMM during four MD steps for a single BN layer with a 60 × 60 supercell (7200 atoms) using the Kim functional versus block size: (*a*) the block size for the basis set, *b*_BF_, is changed and the block size for the wave functions is kept as *b*_WF_ = 6 and (*b*) *b*_WF_ is changed and *b*_BF_ = 13. The relative time is given with respect to the result for *b*_WF_ = 6 and *b*_BF_ = 13. In (*a*), the dashed line is shown to guide the eye for the data obtained for *b*_BF_ divisible by the number of basis functions per atom. The calculations are performed on 192 CPU cores for a DZP basis, *R*_*C*_ = 4 Å, *η* = −5.5 eV.

The dependence of computational time on block size for the Kim functional with DBCSR are presented in figure 7. In the case of the double-zeta polarized (DZP) basis set, each boron and nitrogen atom hosts three localized wave functions and 13 basis functions. Accordingly the computational time drops significantly at block-size values *b*_BF_ for the basis functions divisible by 13 (figure 7*a*). For such block sizes, the computational time grows upon increasing the block size (note that the growth continues beyond the block sizes shown in figure 7*a*) and has the minimum at *b*_BF_ = 13. The wave function block-size *b*_WF_ dependence reaches the minimum at *b*_WF_ = 6 −10. At small *b*_WF_, a fast growth of the computation time is observed. It can be attributed to an increase in the number of non-empty blocks considered upon decreasing the block size. At large *b*_WF_, the computational time also grows but at a slower rate. This dependence can be explained by increasing the number of matrix elements that are stored and explicitly considered in matrix operations. Therefore, we find optimal block sizes both for the wave functions and basis functions of the order of 10. Furthermore, chemical considerations can be exploited when dividing matrices into blocks. Still, the optimal choice of block sizes for complex systems is not straightforward and requires further investigation [26].

**Figure 8.**
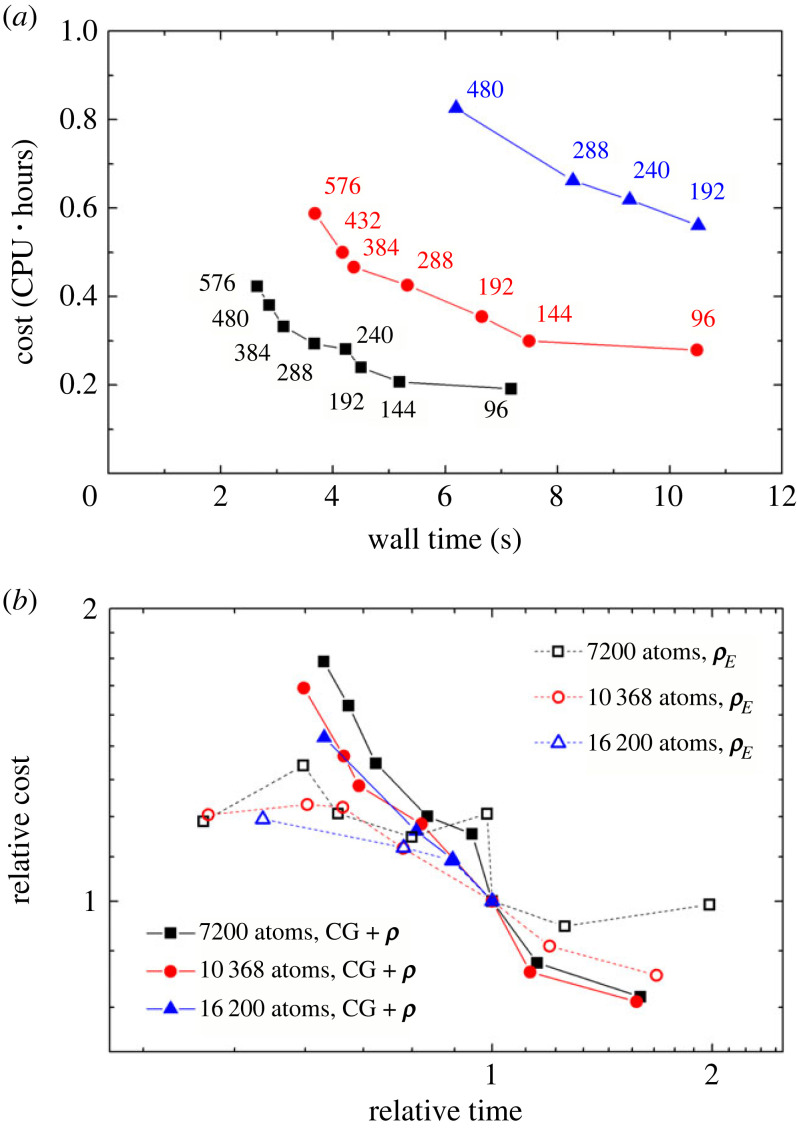
(*a*) Computational cost (in CPU·hours) versus wall time (in s), for one call to the solver library libOMM including one conjugate gradient (CG) iteration and calculation of the density matrix ***ρ***, for different supercells of BN using the Kim functional: (black squares) 60 × 60, (red circles) 72 × 72 and (blue triangles) 90 × 90 (7200, 10 368 and 16 200 atoms, respectively). The number of CPU cores used is indicated. (*b*) Relative computational cost versus relative time for the calls to the solver library including one conjugate gradient iteration and calculation of the density matrix ***ρ*** (closed symbols) or calculation of the energy density ***ρ***_*E*_ (open symbols) for different supercells of BN. Relative values are given with respect to the results for 192 CPU cores. The calculations are for a DZP basis, *R*_*C*_ = 4 Å, *η* = −5.5 eV, *b*_WF_ = 6 and *b*_BF_ = 13.

The CPU scaling of the libOMM solver library in calculations with sparse matrices using DBCSR is shown in figure 8*a*. A similar CPU scaling is observed for systems of different size (figure 8*a*), with different block and basis set sizes. The computational time decreases by a factor of about 2.5 upon doubling the computational cost. Such a speed-up is observed for CG energy minimization and subsequent calculation of ***ρ***. It should be noted, however, that calls to libOMM for calculation of ***ρ***_*E*_ involving only two matrix multiplication operations show much better CPU scaling. This can be appreciated from a twice steeper slope of computational cost versus computational time as compared with the calls for energy minimization and calculation of the density matrix (figure 8*b*). It can, therefore, be expected that the solver parallelization might be further improved via proper code refactoring. The use of OpenMP, GPUs and the library for small matrix multiplication (LIBXSMM) [52] are known to lead to a superior DBCSR performance [25,26], which also requires investigation.

### 4.3. Recommendations for orbital minimization method solver use

The new modular implementation of the OMM solver makes it easier to disentangle technical problems in e.g. parallelization from drawbacks of the OMM itself. Here, we present the first implementation of the solver using external libraries that represents the starting point for further performance improvement and method polishing. Ways to improve the solver performance were mentioned in the previous subsection. We briefly discuss now the drawbacks of the OMM and how they can be addressed.

**Figure 9.**
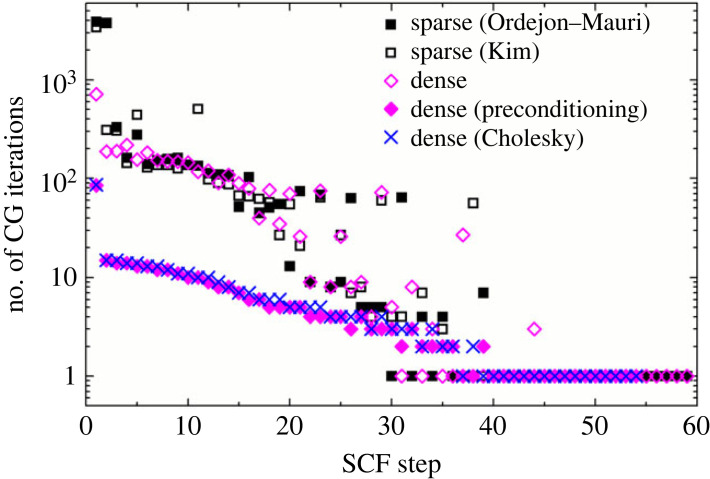
Number of conjugate-gradient (CG) iterations versus number of self-consistent-field (SCF) steps for the calculation of the ground state of a 12 × 12 BN supercell (288 atoms) from scratch using OMM with sparse matrices: (closed black squares) Ordejón–Mauri and (open black squares) Kim functionals, and OMM with dense matrices using the Ordejón–Mauri functional: (open magenta diamonds) plain, (closed magenta diamonds) preconditioned with kinetic-energy scale of *τ*_*T*_ = 10 Ry and (blue crosses) with the Cholesky factorization. The calculations are performed on 96 CPU cores for a DZP basis, *R*_*C*_ = 4 Å, *η* = −5.5 eV, *b*_WF_ = 6 and *b*_BF_ = 13. Linear mixing with a mixing parameter of 0.1 is used.

One of the most important methodological problems of the OMM approach is in the minimization, which can require a large number of CG iterations. As shown in figure 9, the first SCF iteration from scratch is rather costly both for the linear and cubic-scaling OMM. For the linear-scaling methods, the first SCF iteration can include thousands of CG steps, followed by tens of SCF iterations with hundreds of CG steps each. After that each SCF step needs just a few CG iterations, becoming very fast. It should be noted that except for the very first SCF iterations, the linear-scaling and plain cubic-scaling OMM require roughly the same numbers of CG steps. However, kinetic energy preconditioning or Cholesky factorization significantly reduce the number of CG iterations required, with a considerable computational-time reduction (see also figure 5). Therefore, it is always recommended to use either of both ways to deal with kinetic energy ill-conditioning in dense OMM. The extension of these approaches to sparse matrices is not straightforward and requires further investigation.

Also starting from scratch, one can get into regions in parameter space where the energy functional does not have a minimum in the CG line minimization. To avoid this situation, we recommend using a small cut-off radius *R*_*C*,ini_ for the initial guess of wave functions both for linear- and cubic-scaling OMM. It is also recommended to preconverge the ground state using a small linear-mixing parameter. Starting from as low as 0.01 can be required for very large systems. It can then be gradually increased to normal values of 0.1–0.2. After getting close to the ground state, the use of other mixing schemes is possible. If the geometry of the system is far from the optimal one, a reduced step for geometry optimization may also be needed when starting.

**Figure 10.**
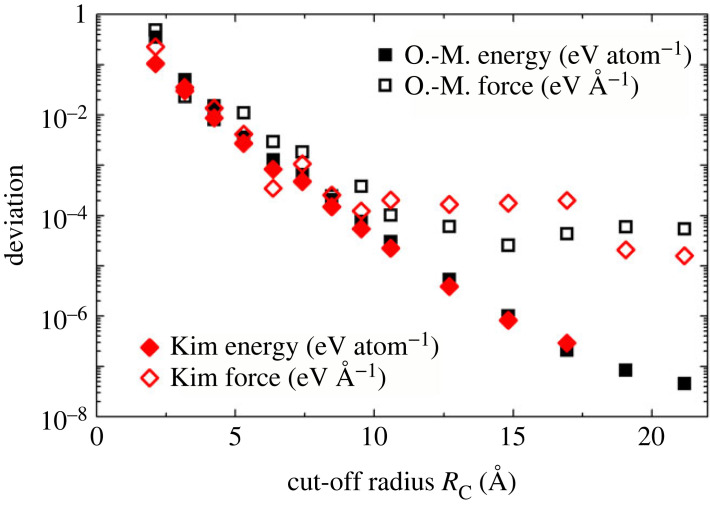
Deviations of energy (in eV atom^−1^, closed symbols) and force (in eV Å^−1^, open symbols) for the 60 × 60 supercell of boron nitride (7200 atoms) with atoms displaced by 0.05 Å from their equilibrium positions from the results for the infinite cut-off radius for the wave functions *R*_*C*_ → ∞ versus cut-off radius *R*_*C*_ (in Å): (black squares) Ordejón–Mauri (O.-M.) and (red diamonds) Kim methods. A DZP basis set is used. The chemical potential for the Kim method is *η* = −5.5 eV. The block size is *b*_WF_ = 6 for the localized wave functions and *b*_BF_ = 13 for the basis functions.

In figure 10, we address the accuracy of force and energy calculations with the Ordejón–Mauri and Kim functionals for BN. The deviation from the results for the wave functions without localization (*R*_*C*_ → ∞) is plotted for different cut-off radii *R*_*C*_. It is seen that for both of the functionals, the accuracy improves upon increasing the cut-off radius in a similar manner. The deviations of the energy and forces within 0.01 eV atom^−1^ and 0.02 eV Å^−1^ are achieved already for the cut-off radius of *R*_*C*_ = 4 Å. These results confirm that for insulating systems with a substantial band gap, it is sufficient to consider cut-off radii of several Å [17,18].

The Ordejón–Mauri and Kim functionals were designed for insulating systems with a substantial gap. For metals, a smearing function needs to be introduced. However, this is not easy since the information on individual Kohn–Sham eigenstates is missing in OMM. An idea for combining OMM with another method resolving eigenstates close to the Fermi level was proposed in [28] but still requires exploration. Note that modelling of metallic systems requires a much more significant computational effort than modelling of insulators [38,39,41].

As for magnetic systems, the OMM calculations can be performed taking into account spin polarization. At each SCF step, the coefficient matrices for spin up and spin down are found sequentially. All the observations for non-spin-polarized systems discussed above still hold in this case.

## 5. Conclusion

We have demonstrated how modularization simplifies the implementation of new solvers in electronic structure codes by revising the OMM solver in the SIESTA code [11–15]. Matrix algebra operations and parallelization are efficiently handled via external libraries. In particular, the implementation benefits from two ESL [9,31] libraries: libOMM [9,27,28,32] and MS [9,29,30,32]. The libOMM library is used to perform the minimization of the energy functional, while the MS library serves as an interface to low-level algebraic routines facilitating switching between different matrix formats. These libraries have been extended to make possible not only cubic-scaling but also linear-scaling OMM calculations for insulating systems with a substantial band gap. Now the energy functional minimization in libOMM can be carried out for sparse matrices with the DBCSR library [24–26], in addition to dense matrices using ScaLAPACK [7]. To facilitate incorporating libOMM into electronic structure codes based on atomic orbitals, MS has been also supplemented with subroutines for matrix format conversion and matrix reading and writing. The solver library libOMM can be easily further developed in the MS language for the implementation of new solvers.

The extended MS and libOMM libraries available through ESL [9,31] can be used for implementation of linear- and cubic-scaling OMM approaches in other codes. The libraries can be used with different types of local basis sets. The only condition for achieving the linear-scaling behaviour is that either the basis functions go to zero beyond some cut-off radius or the elements of the input matrices are filtered with respect to some tolerance to ensure that the matrices are sparse. Note that implementation of custom conversion routines is needed if the matrix format is different from the MS or SIESTA formats.

To test the performance of the new OMM and traditional diagonalization solvers available in SIESTA, large-scale calculations have been performed for a BN layer. When sparse matrices and localized wave functions are used, linear scaling with system size is achieved in practice, as expected. Matrix conversion, reading and writing of restart files, as well as initialization and update of the localized wave functions take a small fraction of the computational time. For the linear-scaling methods that fraction does not depend on system size. The cubic-scaling OMM with kinetic energy preconditioning performs best for small systems, even better than diagonalization. For plain OMM, diagonalization and cubic-scaling OMM with kinetic energy preconditioning, the crossovers with linear-scaling methods are observed at about 300, 700 and 1200 atoms, respectively. The best performance for the linear-scaling OMM with sparse matrices is achieved when the wave functions and basis functions are divided into blocks of sizes around 10, taking into account the chemical structure. The OMM solver is MPI-parallelized. When using the DBCSR library [24–26] for algebraic operations with sparse matrices, the computational time decreases by a factor of 2.5 upon doubling the computational cost. It is expected that CPU scaling can be further improved via refactoring some operations in the libOMM library, using OpenMP and GPUs, etc.

To perform OMM calculations from scratch, it is recommended to start using a small linear-mixing parameter (down to 0.01), a small step for geometry optimization, and cut-off radii for the wave functions of a few Å. For the cubic-scaling OMM, the convergence becomes much faster with kinetic energy preconditioning or Cholesky factorization. The extension of these approaches to sparse matrices demands further investigation.

## Data Availability

The data and relevant code for this research work are stored in GitLab: https://gitlab.com/irina_lebedeva/siesta/-/tree/orderN (SIESTA), https://gitlab.com/ElectronicStructureLibrary/omm-bundle (omm-bundle) and have been archived within the Zenodo repository: https://doi.org/10.5281/zenodo.7781100 [53] (SIESTA), https://doi.org/10.5281/zenodo.7781174 [54] (MatrixSwitch and libOMM). The raw data for tests have been archived within the Mendeley Data repository: https://doi.org/10.17632/c8kz58bg5z.1 [55]. Supplementary material is available online [56].
